# Trust in government regarding COVID-19 and its associations with preventive health behaviour and prosocial behaviour during the pandemic: a cross-sectional and longitudinal study

**DOI:** 10.1017/S0033291721001306

**Published:** 2021-03-26

**Authors:** Qing Han, Bang Zheng, Mioara Cristea, Maximilian Agostini, Jocelyn J. Bélanger, Ben Gützkow, Jannis Kreienkamp, PsyCorona Collaboration, Georgios Abakoumkin, Jamilah H. B. Abdul Khaiyom, Vjollca Ahmedi, Handan Akkas, Carlos A. Almenara, Mohsin Atta, Sabahat Cigdem Bagci, Sima Basel, Edona Berisha Kida, Nicholas R. Buttrick, Phatthanakit Chobthamkit, Hoon-Seok Choi, Mioara Cristea, Sára Csaba, Kaja Damnjanović, Ivan Danyliuk, Arobindu Dash, Daniela Di Santo, Karen M. Douglas, Violeta Enea, Daiane Gracieli Faller, Gavan J. Fitzsimons, Alexandra Gheorghiu, Joanna Grzymala-Moszczynska, Ángel Gómez, Ali Hamaidia, Mai Helmy, Joevarian Hudiyana, Bertus F. Jeronimus, Ding-Yu Jiang, Veljko Jovanović, Željka Kamenov, Anna Kende, Shian-Ling Keng, Tra Thi Thanh Kieu, Yasin Koc, Kamila Kovyazina, Inna Kozytska, Joshua Krause, Arie W. Kruglanski, Anton Kurapov, Maja Kutlaca, Nóra Anna Lantos, Edward P. Lemay, Cokorda Bagus J. Lesmana, Winnifred R. Louis, Adrian Lueders, Najma Iqbal Malik, Anton P. Martinez, Kira O. McCabe, Jasmina Mehulić, Mirra Noor Milla, Idris Mohammed, Erica Molinario, Manuel Moyano, Hayat Muhammad, Silvana Mula, Hamdi Muluk, Solomiia Myroniuk, Reza Najafi, Claudia F. Nisa, Boglárka Nyúl, Paul A. O'Keefe, Jose Javier Olivas Osuna, Evgeny N. Osin, Joonha Park, Gennaro Pica, Antonio Pierro, Jonas H. Rees, Elena Resta, Marika Rullo, Michelle K. Ryan, Adil Samekin, Pekka Santtila, Edyta Sasin, Birga M. Schumpe, Heyla A. Selim, Michael Vicente Stanton, Wolfgang Stroebe, Samiah Sultana, Robbie M. Sutton, Eleftheria Tseliou, Akira Utsugi, Caspar J. Van Lissa, Kees Van Veen, Michelle R. vanDellen, Alexandra Vázquez, Robin Wollast, Victoria Wai-lan Yeung, Somayeh Zand, Iris Lav Žeželj, Andreas Zick, Claudia Zúñiga, N. Pontus Leander

**Affiliations:** 1 School of Psychological Science, University of Bristol, Bristol, UK; 2 Ageing Epidemiology Research Unit, School of Public Health, Imperial College London, London, UK; 3 Department of Psychology, Heriot Watt University, Edinburgh, UK; 4 Department of Psychology, University of Groningen, Groningen, Netherlands; 5 Department of Psychology, New York University Abu Dhabi, Abu Dhabi, United Arab Emirates

## Abstract

**Background:**

The effective implementation of government policies and measures for controlling the coronavirus disease 2019 (COVID-19) pandemic requires compliance from the public. This study aimed to examine cross-sectional and longitudinal associations of trust in government regarding COVID-19 control with the adoption of recommended health behaviours and prosocial behaviours, and potential determinants of trust in government during the pandemic.

**Methods:**

This study analysed data from the PsyCorona Survey, an international project on COVID-19 that included 23 733 participants from 23 countries (representative in age and gender distributions by country) at baseline survey and 7785 participants who also completed follow-up surveys. Specification curve analysis was used to examine concurrent associations between trust in government and self-reported behaviours. We further used structural equation model to explore potential determinants of trust in government. Multilevel linear regressions were used to examine associations between baseline trust and longitudinal behavioural changes.

**Results:**

Higher trust in government regarding COVID-19 control was significantly associated with higher adoption of health behaviours (handwashing, avoiding crowded space, self-quarantine) and prosocial behaviours in specification curve analyses (median standardised *β* = 0.173 and 0.229, *p* < 0.001). Government perceived as well organised, disseminating clear messages and knowledge on COVID-19, and perceived fairness were positively associated with trust in government (standardised *β* = 0.358, 0.230, 0.056, and 0.249, *p* < 0.01). Higher trust at baseline survey was significantly associated with lower rate of decline in health behaviours over time (*p* for interaction = 0.001).

**Conclusions:**

These results highlighted the importance of trust in government in the control of COVID-19.

## Introduction

To address the growing public health crisis created by the COVID-19 pandemic, governments across the world need to play an essential role in the prevention and control of the disease while mitigating its economic impact. Numerous countries have introduced responsive measures and regulations to prevent disease transmission [e.g. social distancing, handwashing, self-isolation (World Health Organization, 2020)] and stabilise the economy. However, effective implementation of these measures depends on a high level of compliance and support from the public (Anderson, Heesterbeek, Klinkenberg, & Hollingsworth, 2020). Emerging theoretical and empirical evidence suggests that trust in government is crucial to public's compliance with social policies that rely on their behavioural responses (Chanley, Rudolph, & Rahn, 2000; Lau et al., 2020; OECD, 2017). As such, understanding the association between trust in government and the adoption of preventive behaviours and exploring various determinants of trust in government during the pandemic are important for the control of COVID-19.

Trust in government represents the confidence or satisfaction of people with government performance and the perceived credibility of government (Bouckaert & Van de Walle, 2003; Christensen & Lægreid, 2005; Uslaner, 2018; Zmerli & Van der Meer, 2017). It has been identified as a cornerstone of the political system, particularly in crises such as natural disasters, economic crises, or pandemics (Rodriguez, Donner, & Trainor, 2018). Trust in government produces spontaneous sociability, which in turn leads to cooperative, altruistic and extraterritorial behaviours in social activities (Fukuyama, 1995; Hetherington, 1998). Previous studies demonstrated that the higher level of trust in government was associated with greater willingness to follow a range of government recommendations and prosocial behaviours, such as adopting preventive behaviours to avoid the swine flu (Rubin, Amlot, Page, & Wessely, 2009), getting vaccinated against seasonal influenza (Verger, Bocquier, Vergelys, Ward, & Peretti-Watel, 2018), and making economic sacrifice for the environment (Taniguchi & Marshall, 2018). There has been some preliminary evidence linking public trust to compliance with government guidelines at the early stage of COVID-19 pandemic (Bargain & Aminjonov, 2020; Devine, Gaskell, Jennings, & Stoker, 2020; Freeman et al., 2020; Goldstein & Wiedemann, 2020; Olsen & Hjorth, 2020; Schmelz, 2021). However, these studies were generally limited by small sample size, data from single country, restricted to early stage of pandemic, and the cross-sectional design. The only longitudinal study is a multi-wave survey of 633 participants in Singapore during January−April 2020 (Lim et al., 2021), which showed trust in government communication was positively associated with likelihood of adopting protective behaviour; but that study did not investigate the effect of trust on longitudinal behavioural changes. Moreover, prosocial behaviour has been neglected in previous COVID-19 research, which is important because social support and volunteering could facilitate the prevention and treatment of coronavirus.

Given the importance of maintaining public trust during the pandemic, there is an urgent need to identify the determinants of trust in government regarding the ability and efficacy of COVID-19 control. The Organization for Economic Co-operation and Development (OECD, 2017) pointed out that reliability, responsiveness, openness, better regulation, fairness, and inclusive policy making are key areas for governments to gain public trust. In the context of the current pandemic, better regulation and organisation of government in the design and implementation of responsive measures could increase public support and trust in government (Van Bavel et al., 2020). In addition, the lack of transparency and timely and accurate communication of government has been identified as a major element that has caused the decline of trust in government (O'Malley, Rainford, & Thompson, 2009; Welch, Hinnant, & Moon, 2005). Furthermore, trust in government is influenced by the performance of the national economy and citizens' evaluations of the economy (Miller & Borrelli, 1991). Finally, lack of perceived fairness which refers to being treated equally as other people in society could also lead to distrust in government, especially during crises (Meredith, Eisenman, Rhodes, Ryan, & Long, 2007).

Based on the theoretical background and empirical evidence, we conducted one of the first large-scale international surveys focusing on trust in government and longitudinal behavioural responses from the public during the unprecedented COVID-19 pandemic. The aim of this study was threefold: (1) to examine the cross-sectional associations between trust in government on COVID-19 and the adoption of health and prosocial behaviours that are crucial for pandemic control; (2) to explore potential determinants of the COVID-19-related trust in government, including government regulation, clear information or knowledge on COVID-19, economic status, and perceived fairness during the pandemic; and (3) to examine the associations between public trust in government on COVID-19 at baseline survey and changes in health and prosocial behaviours during follow-up.

## Methods

### Data source

This study was based on cross-sectional and longitudinal data from the PsyCorona Survey on COVID-19 (Project website: https://psycorona.org). This 20-minute web-based survey, translated into 30 languages, aimed to investigate the psychological and behavioural impact of the coronavirus spread. During 10 April to 11 May 2020, the PsyCorona Survey actively recruited 23 733 participants from 23 countries. Participants were sampled online through Qualtrics' panel management service, so that they are representative of the country's general population in terms of gender and age. About 1000 participants were selected for each of the 23 countries (Argentina, Australia, Brazil, Canada, France, Germany, Greece, Indonesia, Italy, Japan, Netherlands, Philippines, Romania, Russia, Saudi Arabia, Serbia, South Africa, South Korea, Spain, Turkey, the United Kingdom, Ukraine, and the United States of America). After the baseline survey, participants were invited to complete multiple waves of weekly or monthly follow-up surveys on a voluntary basis to measure the changes of psychological and behavioural responses to COVID-19 over time.

PsyCorona Survey was approved by the Ethical Committee of the University of Groningen (study code: PSY-1920-S-0390) and New York University Abu Dhabi (study code: HRPP-2020-42). All participants gave informed consent before taking the survey.

### Measures

This study focused on measures of trust in government regarding COVID-19 control (three items, Cronbach's *α* = 0.754), adoption of preventive health behaviours (three items, Cronbach's *α* = 0.795), and willingness to engage in COVID-19-related prosocial behaviours (four items, Cronbach's *α* = 0.801; Table 1). Of the three items on trust in government, one directly measured trust in country government to take the right response measures (rated in 5-point Likert scale from 1 (not at all) to 5 (a great deal)), two measured trust of country's ability to fight COVID-19 or its economic consequences (7-point Likert scale from −3 (strongly disagree) to 3 (strongly agree)). Since the government in all 23 sample countries plays a major role in pandemic control, public's trust in country could reflect their trust in government towards COVID-19. All three items on health behaviour and four items on prosocial behaviour were in 7-point Likert scale from −3 (strongly disagree) to 3 (strongly agree).

**Table 1.** Items on trust in government, health behaviour, and prosocial behaviour with possible model specifications

Constructs	Items	Analytical decisions
Trust in government regarding COVID-19	In general, how much do you trust the government of your country to take the right measures to deal with the coronavirus pandemic?	Each item individually; mean of the three items; the first principal component of three items (which explains 68% of total variance and 59%, 72% and 72% of variance of each item).
	I think that this country is able to fight the coronavirus.	
	I think that this country is able to fight the economic and financial consequences of coronavirus.	
Personal health behaviour	To minimize my chances of getting coronavirus, I wash my hands more often.	Each item individually; mean of the three items; the first principal component of three items (which explains 69% of total variance and 74%, 82% and 62% of variance of each item).
	To minimize my chances of getting coronavirus, I avoid crowded spaces.	
	To minimize my chances of getting coronavirus, I put myself in quarantine.	
Prosocial behaviour	I am willing to help others who suffer from coronavirus.	Each item individually; mean of the four items; the first principal component of four items (which explains 63% of total variance and 62%, 66%, 71% and 52% of variance of each item).
	I am willing to make donations to help others that suffer from coronavirus.	
	I am willing to protect vulnerable groups from coronavirus even at my own expense.	
	I am willing to make personal sacrifices to prevent the spread of coronavirus.	

All of the above-mentioned items were included in the baseline survey. Besides, the three items of preventive health behaviours and the first and third items on prosocial behaviours were repeatedly measured in Wave 4 (launched on 25 April 2020), Wave 8 (23 May 2020), Wave 11 (13 June 2020), Wave 12 (13 July 2020), Wave 13 (13 August 2020) and Wave 14 (13 September 2020) of the follow-up surveys.

In addition, information on a set of covariates were collected in the baseline survey, including age group, gender, education level, citizenship, religion, close relationship with infected patients, employment status, personal financial strain, perceived fairness, perceived knowledge on COVID-19, clear message received on COVID-19, and government being well-organised in response to the pandemic. Details of relevant items are displayed in online Supplementary Table S1.

### Eligible participants

A total of 24 261 participants selected from 23 countries completed the baseline survey. Participants with any missing values in items on trust in government, health and prosocial behaviours, age group, and gender were excluded, which resulted in a sample of 23 733 participants for the cross-sectional analyses (sample size of each country varies from 738 to 1159). For the longitudinal analyses, 7785 participants from both convenience sample and representative sample of the PsyCorona study in these 23 countries who have completed both baseline survey and at least one follow-up survey (Wave 4, 8 or 11–14) were included. Complete case analysis was used to deal with missing values on covariates in relevant analyses (each covariate had 0 to <1% missing values).

### Statistical analysis

The statistical methods used in the analyses, corresponding to the three research aims mentioned in the Introduction, are described separately below (also shown in the directed acyclic graph in online Supplementary Fig. S1). For aim 1, specification curve analysis (SCA) was used to examine the concurrent associations of trust in government regarding COVID-19 with health behaviours and prosocial behaviours separately based on the baseline survey. For aim 2, structural equation model (SEM) was used to explore potential determinants of trust in government at the baseline survey. For aim 3, multilevel linear regressions were used to examine associations between baseline trust in government and longitudinal behavioural changes as measured in follow-up surveys.

#### Specification curve analysis

Given the fact that there are multiple items on each measure and various analytical options regarding covariate adjustment, it is difficult to select one optimal model specification for testing the association of trust in government with health and prosocial behaviours without introducing subjective bias. Therefore, we used specification curve analysis (Orben & Przybylski, 2019; Simonsohn, Simmons, & Nelson, 2015), a recently developed robust statistical method that considers all reasonable model specifications to avoid subjective analytical decisions. Based on multilevel linear regressions (Bingenheimer & Raudenbush, 2004) with behaviour measures as dependent variable and country-level intercept as random effect, multiple analytical options were tested. For each of the three constructs (i.e. trust, health behaviour, prosocial behaviour), relevant items were tested individually and in combination as mean score or through principal component analysis (PCA, Table 1). The results of PCA showed a single principal component with eigenvalue greater than 1 for all three constructs, which explains most variations of corresponding items. In addition, to account for potential confounding bias, three specifications were considered: no covariates, only adjusting for basic demographics (age, gender, and education level), or adjusting for a full set of covariates as mentioned above. After combining three model specification factors (independent variable, dependent variable, and covariate adjustment), the total numbers of model specifications are 75 for trust in government and adoption of health behaviour (5 for trust × 5 for health behaviour × 3 for covariates), and 90 for trust in government and prosocial behaviour (5 for trust × 6 for prosocial behaviour × 3 for covariates).

After implementing all model specifications, the median standardised *β* and median standard error (s.e.) were used as summary statistics. Due to missing values in covariates, the sample sizes were 23 733, 23 693, and 23 406 for models with no covariates, with adjustment for basic demographics, and fully adjusted models in SCA.

For the overall statistical inferences of SCA, a bootstrapping technique was used to perform joint significance tests. A pseudo-dataset was created by replacing the original dependent variable with the residuals in each model specification, where the null hypothesis holds (i.e. true *β* = 0). Using random sampling with replacement, 1000 bootstrapped datasets of equal size as the pseudo-dataset were generated, on which 1000 repeated SCAs were conducted. The null hypothesis was rejected if the possibility of re-sampled median standardised *β* being larger in magnitude than observed value in original SCA was below 0.05, or the possibility of getting an equal or larger number of significant tests as in original SCA by chance was below 0.05.

#### SEM analysis

**Fig. 1.**
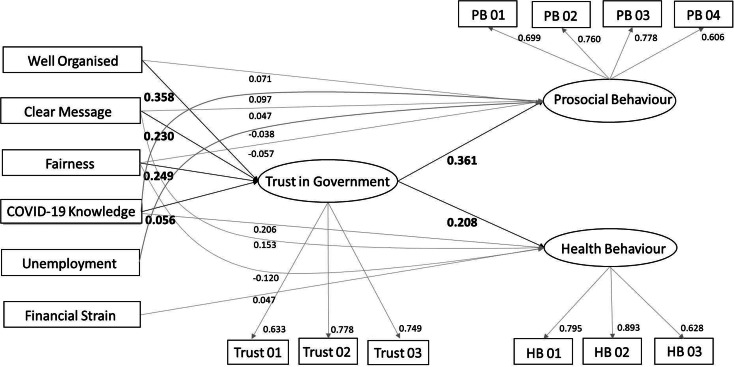
Results of SEM analysis. *Note.* Only paths with significant regression coefficients (*p* < 0.05) are plotted. Standardised *β* coefficients are displayed on the lower-right side of the corresponding paths. Trust 01-03 refer to the three items of trust in government; HB 01-03 refer to the three items of health behaviour; PB 01-04 refer to the four items of prosocial behaviour.

Within the generalised SEM, associations between hypothesised determinants of trust in government, latent variable of trust in government, and latent variables of health and prosocial behaviours were estimated based on multilevel linear regressions, with country-level intercept as random effects (Fig. 1). Standardised regression coefficients were estimated and tested in all regression models. Multiple fitting indices were calculated to evaluate the overall model fit.

Hypothesised determinants of trust in government regarding pandemic control include employment status (employed, not employed, or other), personal financial strain (in 5-point Likert scale), perceived fairness (in 5-point Likert scale), knowledge on COVID-19 (in 5-point Likert scale), receiving clear messages on coping with COVID-19 (in 6-point Likert scale), and government being well-organised in response to pandemic (in 6-point Likert scale; online Supplementary Table S1). In addition, the SEM also serves as a complementary analysis to SCA by estimating the associations between latent variables of trust in government and willingness to adopt health and prosocial behaviours.

#### Longitudinal analysis with multilevel linear regressions

Multilevel linear regression model with subject-level random intercepts was used to analyse the within-subject changes of COVID-19-related behaviours over time. In the analysis of changes in health behaviours, the repeated measurement of mean score of three health behaviour items was modelled as dependent variable; the baseline mean score of three trust in government items, days since baseline survey, and an interaction term of these two variables were modelled as independent variables; age, gender and education level were controlled for as covariates. The interaction term (baseline trust × days) reflects the influence of baseline trust level on the subsequent daily changes in health behaviours. Similarly, the changes of prosocial behaviours were analysed in a separate model, with the repeated measurement of mean score of prosocial behaviour items as dependent variable.

We also conducted a subgroup analysis by national income level (World Bank, 2020) of the 23 countries (14, 7 and 2 countries are high, upper-middle and lower-middle income countries) to explore whether the associations between trust in government and behaviours vary across country categories. No conventional effect size was computed in this study because all analyses were based on multilevel linear models with random effects, for which the standardised *β* has been recommended as one of the optimal effect sizes to reflect the magnitude of fixed effects (Lorah, 2018).

All statistical analyses were conducted using R software (version 4.0.0). Codes for SCA were adapted from functions developed by Orben and Przybylski (2019). The sem function of lavaan package was used for the SEM analysis. The lmer function of lme4 package was used for the longitudinal analysis. All statistical tests are two-sided. Where applicable, *p* < 0.05 indicates statistical significance.

## Results

### Population characteristics and country-level descriptions

Of the 23 733 participants from 23 countries who are representative of the country population in terms of age and gender, 51% are women, 32%, 54%, or 14% are aged between 18–34, 35–64, or over 65 years, and 59%, 29%, or 12% have education level below, equivalent, or above Bachelor's degree. Of the 7785 participants with follow-up data, 62% are women; 25%, 56%, or 19% are aged between 18–34, 35–64, or over 65 years old; and 52%, 26%, or 22% have education level below, equivalent, or above Bachelor's degree.

**Fig. 2.**
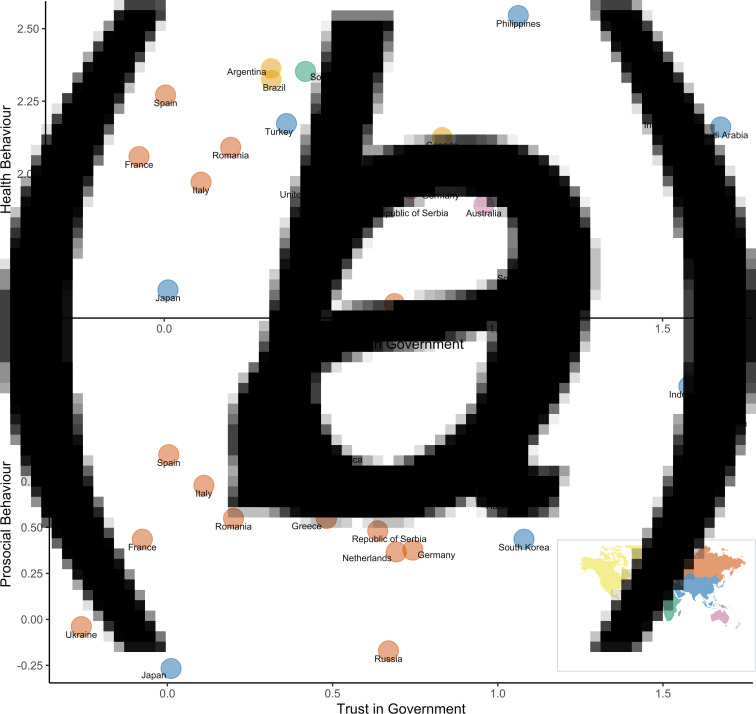
Scatter plots of country-level mean values of health behaviour items (*a*) and prosocial behaviour items (*b*) against mean values of trust in government items. *Note.* Data on 23 countries from the five continents are displayed as circles in each plot. Each colour corresponds to a particular continent. Three items on trust in government were harmonised into 7-point scale from −3 (strongly disagree) to 3 (strongly agree); three items on health behaviour and four items on prosocial behaviour were in similar scale from −3 to 3.

The scatter plots of country-level summary statistics at baseline survey showed a positive correlation between the country-level mean values of trust in government and country-level prosocial behaviour, whereas no clear trend of correlation was observed for the country-level health behaviour (Fig. 2).

### Specification curve analysis for associations of trust in government with health behaviour and prosocial behaviour

**Fig. 3.**
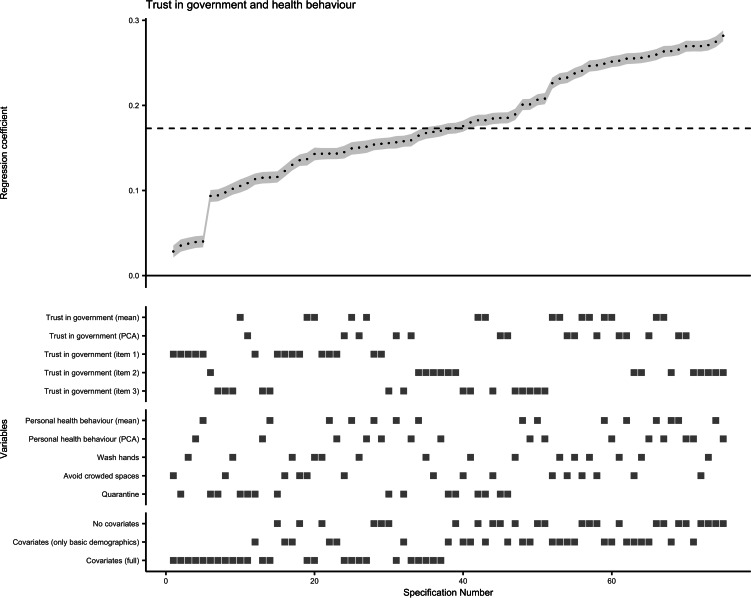
Results of specification curve analysis for trust in government and adoption of personal health behaviour. *Note.* The standardised *β* coefficients for the association of trust in government with health behaviour obtained from all 75 specifications (listed on the *x* axis) are plotted at the upper half of the graph. Each point represents the standardised *β* coefficient of one specification, and the error bar (in grey) represents the corresponding standard error. The dashed line indicates the median standardised *β* coefficient (median standardised *β* = 0.173, median standard error = 0.007, median sample size = 23 693). At the lower half of the graph, the corresponding specifications for each level of the three model specification factors are displayed as squares.

All 75 model specifications for multilevel linear regression of COVID-19-related health behaviour on trust in government revealed significant positive association (maximum *p* for single test = 6 × 10^−5^). The standardised *β* coefficients and s.e. obtained for this association from all specifications are plotted in Fig. 3, with a median standardised *β* of 0.173 (median s.e. = 0.007). Similarly, the median standardised *β* of 15 specifications with the single-item direct measure of trust in government as independent variable was 0.123 (median s.e. = 0.007). The results of the bootstrapped test supported the overall association between trust in government regarding pandemic control and compliance with recommended health behaviours. The probability of having a median standardised *β* > 0.173 or <−0.173 (i.e. stronger than in original SCA), or getting 75 significant tests by chance was below 0.001 when the null hypothesis is true.

Furthermore, the SCA plot visualised the influences of different analytical options on the effect estimates (Fig. 3). The health behaviour of self-quarantine had a slightly weaker association with trust (median standardised *β* = 0.156, median s.e. = 0.007) than washing hands more frequently or avoiding crowded space (median standardised *β* = 0.180 or 0.176, median s.e. = 0.007). Not adjusting for covariates or only adjusting for basic demographics yielded similar effect estimates (median standardised *β* = 0.208 or 0.201, median s.e. = 0.007 or 0.006), whereas adjusting for a full set of covariates showed a weaker independent effect of trust in government on adoption of health behaviour (median standardised *β* = 0.115, median s.e. = 0.007).

**Fig. 4.**
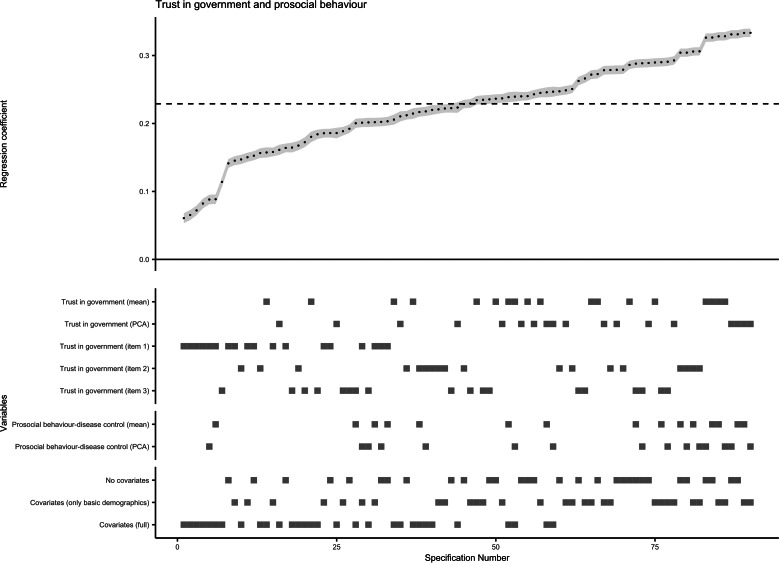
Results of specification curve analysis for trust in government and adoption of prosocial behaviour. *Note.* The standardised *β* coefficients for the association of trust in government with prosocial behaviour obtained from all 90 specifications (listed on the *x* axis) are plotted at the upper half of the graph. Each point represents the standardised *β* coefficient of one specification, and the error bar (in grey) represents the corresponding standard error. The dashed line indicates the median standardised *β* coefficient (median standardised *β* = 0.229, median standard error = 0.006, median sample size = 23 693). At the lower half of the graph, the corresponding specifications for each level of the three model specification factors are displayed as squares; the four individual items of prosocial behaviour were omitted due to the figure size.

As for the association between trust in government and COVID-19-related prosocial behaviour, all 90 model specifications of multilevel linear regression revealed significant positive association (maximum *p* for single test = 2 × 10^−16^). The median standardised *β* coefficient obtained from all specifications was 0.229 (median s.e. = 0.006; Fig. 4). The bootstrapped tests showed that, when the null hypothesis is true, the possibility of having a median standardised *β* > 0.229 or <−0.229, or getting 90 significant tests by chance was below 0.001. Therefore, the null hypothesis was rejected and the existence of a positive association was confirmed.

As shown in Fig. 4, trust of country's ability to fight the coronavirus or economic consequences had a stronger association with adoption of prosocial behaviour (median standardised *β* = 0.225 or 0.226, median s.e. = 0.006) than trust in country government to take right response measures (median standardised *β* = 0.151, median s.e. = 0.007). Similar to the situation in SCA for health behaviour, controlling for a full set of covariates resulted in a weaker independent effect of trust in government on prosocial behaviour (median standardised *β* = 0.182, median s.e. = 0.007). The subgroup analysis by country categories showed consistent SCA results across low-middle, upper-middle and high-income countries.

### SEM for potential determinants of COVID-19-related trust in government and behaviour

After establishing the associations between trust in government and health and prosocial behaviours, we further built an integrated model with multilevel SEM to explore potential determinants of trust in government in the context of COVID-19 control (Fig. 1). After controlling for potential confounding variables (age, gender, education level, religion, citizenship, and close relationship with infected patients), the overall trust in government regarding pandemic control was positively associated with willingness to adopt recommended health and prosocial behaviours (standardised *β* = 0.208 and 0.361; *p* < 0.001), which further supported the findings from the SCA models (Fig. 1). As for the hypothesised determinants, governments being well-organised in response to the pandemic, more fairness, more clear messages received on coping with COVID-19, and more knowledge on COVID-19 were associated with higher level of overall trust in government (standardised *β* = 0.358, 0.249, 0.230, and 0.056; *p* < 0.01). In contrast, employment status and personal financial strain were not significantly associated with overall trust in government regarding pandemic control (*p* > 0.05). The fitting indices demonstrated a good fit between this SEM and the data (root mean square error of approximation = 0.021, standardised root mean square residual = 0.020, comparative fit index = 0.935). The sensitivity analyses without adjusting for potential confounding variables or using the single-item direct measure of trust in government yielded similar results.

Furthermore, perceived knowledge and message clarity on COVID-19, fairness, and personal financial strain also had direct associations with willingness to adopt recommended health behaviour (standardised *β* = 0.206, 0.153, −0.120, and 0.047; *p* < 0.001). Governments being well-organised had direct association with prosocial behaviour (standardised *β* = 0.071; *p* < 0.01) but not health behaviour (*p* > 0.05). Besides, perceived knowledge and message clarity on COVID-19, fairness, and unemployment were directly associated with prosocial behaviour (standardised *β* = 0.097, 0.047, −0.057, and −0.038; *p* < 0.05).

### Longitudinal analysis for associations of baseline trust in government with subsequent changes in health behaviour and prosocial behaviour

**Fig. 5.**
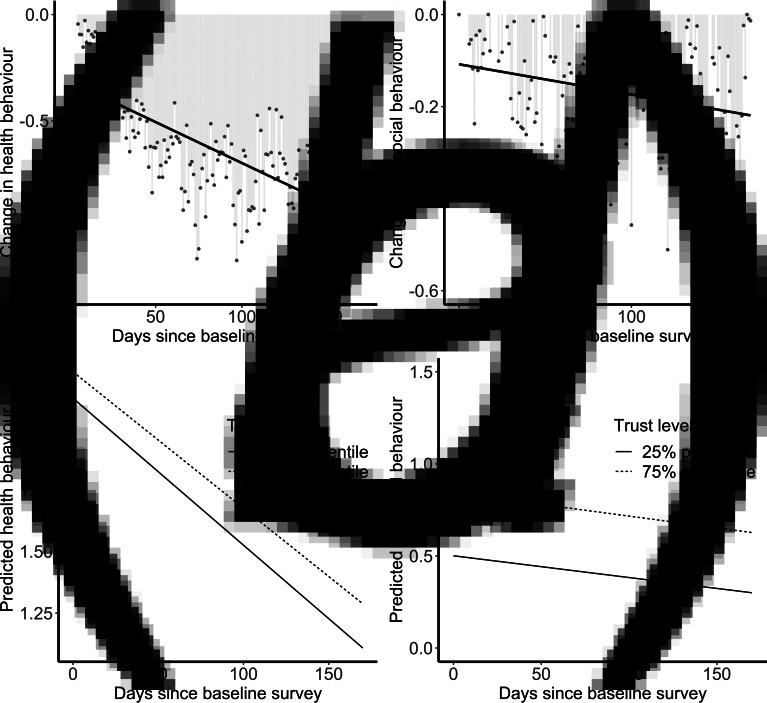
Longitudinal changes in health and prosocial behaviours and the influences of baseline trust in government. *Note.* The average differences between follow-up survey and baseline survey in mean score of three health behaviour items (*a*) and mean score of two prosocial behaviour items (*b*) were plotted against time since baseline survey. A negative value indicates a decline of behaviour adoption at follow-up survey. Items on behaviour were in a 7-point scale from −3 (strongly disagree) to 3 (strongly agree). The lower half of the graph shows the predicted values (marginal means) of mean score of health behaviours (*c*) and mean score of prosocial behaviours (*d*) across time, obtained from multilevel linear regressions. The solid line in (*c*) and (*d*) represents the predicted values at the 25% percentile of baseline mean score of trust in government (−0.5 in a −3 to 3 scale); the dashed line represents the predicted values at the 75% percentile of baseline mean score of trust in government (1.5 in a −3 to 3 scale).

The scatter plots of average differences between follow-up survey and baseline survey in mean score of health behaviours (Fig. 5*a*) and prosocial behaviours (Fig. 5*b*) showed a trend of decline in both behaviours. The results of multilevel linear regressions demonstrated that higher baseline trust was significantly associated with lower rate of decline in adoption of health behaviours over time (*β* for baseline trust × days = 0.021; *p* for interaction = 0.001; Fig. 5*c*). The interaction term was not significant for prosocial behaviour (*p* > 0.05), but the main effects of baseline trust and days since baseline survey (per 100 days) on longitudinal prosocial behaviour were significant (*β* = 0.171 and −0.121; *p* < 0.001; Fig. 5*d*). Sensitivity analyses modelling each behavioural item as dependent variable, not adjusting for covariates or adjusting for the additional covariates yielded similar results. The subgroup analysis by country categories showed consistent results except that the interaction term for health behaviour was significant in upper-middle and low-middle income country but not in high-income country.

## Discussion

In this first large-scale cross-country study focusing on COVID-19-related trust in government, we found a robust relationship between trust and personal preventive behaviour. A higher level of trust in government regarding COVID-19 control was significantly associated with higher compliance with measures of frequent handwashing, avoiding crowded spaces, and social isolation/quarantine. This result is consistent with previous findings that public trust was associated with adherence to public health interventions (Goold, 2002; Meredith et al., 2007; Mohseni & Lindstrom, 2007; O'Malley, Sheppard, Schwartz, & Mandelblatt, 2004; Salmon, Dudley, Glanz, & Omer, 2015). Two representative surveys in Liberia and Congo during the Ebola outbreaks also indicated that trust in government was positively related to compliance with disease control measures (Blair, Morse, & Tsai, 2017) or adoption of personal preventive behaviours (e.g. keeping social distance and accepting Ebola vaccines) (Vinck, Pham, Bindu, Bedford, & Nilles, 2019).

In addition, the results showed a significant positive association between trust in government and willingness to engage in prosocial behaviours that aid the control of COVID-19 pandemic. This is in line with a number of previous studies where higher levels of trust in government are related to more support for public welfare policies and willingness to sacrifice personal material interests (Hetherington & Husser, 2012; Rudolph & Evans, 2005; Scholz & Lubell, 1998). As hypothesised, in a low-trust environment, citizens will prioritise immediate and partial benefits (Gyorffy, 2006), whereas high levels of trust towards the long-term benefits of public policies could produce spontaneous sociability that motivates the self-sacrifice of some immediate benefits (Fukuyama, 1995; Murphy, 2004). Our study further affirmed this statement in the context of the current public health crisis. Moreover, we found that the trust of fighting the economic consequences was also associated with adoption of prosocial behaviour, which is plausible because the reduction in people's financial concern may increase their altruistic behaviours such as donation.

Our longitudinal analysis showed a significant moderating effect of trust in government at baseline survey on the rate of health behaviour changes over time. High trust in government could prevent the public compliance with recommended health behaviours from rapid decline due to fatigue or reduced attention. The reduced acceptance of official information caused by distrust in government fosters the spread of fake news and misinformation (Garry, Ford, & Johns, 2020; Lau et al., 2020; Salali & Uysal, 2020), which could substantially affect the formation and the maintaining of people's health behaviours. It has been argued that limited trust in government could make the control of COVID-19 more difficult, especially in low- and middle-income countries (Lloyd-Sherlock, Ebrahim, Geffen, & Mckee, 2020). In line with this concern, our subgroup analysis revealed that the moderating effect of trust in government is more manifest in middle-income countries instead of high-income countries. Although we did not observe similar moderating effect of trust on changes in prosocial behaviour, the main effect showed that people with higher baseline trust had a sustained higher level of prosocial behaviour throughout the follow-up period compared to those with lower baseline trust.

In the context of this worldwide pandemic, the international cooperation between governments and people all over the world is the key to stop the spreading of the coronavirus. Both personal preventive health behaviour and the prosocial behaviour that offers support for others are essential for fighting the COVID-19. In this regard, building public trust in government regarding disease control could serve as an effective strategy to achieve a better cooperation and compliance with COVID-19-related policies and interventions, and ultimately improve the prevention and control of this disease.

Given the importance of trust in government on COVID-19, we further explored its determinants which are modifiable for a better translation into public policies. The results showed that government that was perceived as well organised in response to COVID-19, clear messages and perceived knowledge on COVID-19, and perceived fairness were positively associated with trust in government. This implies that clear information and unambiguous health instructions that represent government transparency and effective communication are important in terms of maintaining public trust (Norris, 2002; Worthy, 2010). The result on perceived fairness is in line with previous studies that linked feelings of social inequality with less trust in government or public health institutes (Alsan & Wanamaker, 2018; Meredith et al., 2007). Therefore, the fairness in the pandemic control should be treated with caution.

The strength of this study lies in its large samples from diverse geographic regions worldwide (both high- and middle-income countries), which is especially important in the investigation of trust in government. This large-scale quantitative study provided empirical evidence on the behavioural influences of public trust across a relatively long period of time (April−October 2020), in the context of the ongoing COVID-19 crisis. Moreover, this study collected sufficient information on potential confounding variables, as well as potential determinants of trust in government to shed light on practical implications.

Nevertheless, this study has several limitations. Although the age and gender distributions of included participants within each country matched their national population structure, this is not necessarily a fully representative sample. Nevertheless, this carefully designed suboptimal online sampling strategy seems to be the most practical and efficient way during the ongoing pandemic. Despite the examinations of both cross-sectional and longitudinal data, causal inferences for the hypothesised determinants of trust in government and its behavioural impact on pandemic control need to be made with caution given the observational nature of this study. Furthermore, a more detailed investigation on different aspects or dimensions of COVID-19-related trust in government or health institutes, such as the trust of detection capacity, clinical pathways, or vaccination, is needed for a comprehensive understanding of this topic.

In conclusion, this study demonstrates that stronger trust in government on COVID-19 control is associated with higher willingness to adopt recommended health and prosocial behaviours and slower decline in the adoption of health behaviours over time. In addition, governments being better organised in response to the pandemic, more unambiguous messages received and perceived knowledge on COVID-19, and higher perceived fairness are associated with higher level of trust in government. Relevant public policies targeting to improve public trust in fighting the coronavirus and dealing with secondary consequences could hugely facilitate the control of the pandemic.

## Supplementary material

For supplementary material accompanying this paper visit http://dx.doi.org/10.1017/S0033291721001306. click here to view supplementary material
