# Supplementary material for: Trust in government regarding COVID-19 and its associations with preventive health behaviour and prosocial behaviour during the pandemic: a cross-sectional and longitudinal study
Source: Psychol Med. 2021 Mar 26:1–11. doi: 10.1017/S0033291721001306 (PMC8144822; doi:10.1017/S0033291721001306)

**Supplementary Content**

Supplementary Table S1. Items of covariates in the PsyCorona survey

Supplementary Figure S1. Directed acyclic graph of three research aims and corresponding statistical approaches

**Supplementary Table S1**

*Items of covariates in the PsyCorona survey*

| **Covariates** | **Items** | **Scale/options** |
| --- | --- | --- |
| Age | What is your age? | 18-24; 25-34; 35-44; 45-54; 55-64; 65-74; 75-84; 85+ |
| Gender | What is your gender? | Female; male; other |
| Education level | What is your highest level of education? | Primary education; general secondary education; vocational education; higher education; Bachelor’s degree; Master’s degree; PhD degree |
| Religion | Are you religious? | Yes; no |
| Citizenship | Are you a citizen of this country? | Yes; no |
| Employment status | Which of the following categories best describes your employment status during the last week? | Employed; not employed; other |
| Personal financial strain | Agree or disagree: - I am financially strained. | 5-point scale from -2 (strongly disagree) to 2 (strongly agree) |
| Close relationship with infected patient | Do you personally know anyone who currently has coronavirus? | Myself; a member of my family; a close friend; someone I know; someone else; I do not know anyone |
| Knowledge about COVID-19 | How knowledgeable are you about the recent outbreak of Covid-19, commonly referred to as the Coronavirus, in this country? | 5-point scale from 1 (not at all knowledgeable) to 5 (extremely knowledgeable) |
| Clear message on coping with COVID-19 | To what extent are you getting clear, unambiguous messages about what to do about the Coronavirus? | 6-point scale from 1(messages are completely unclear/ ambiguous) to 6 (messages are very clear/ unambiguous) |
| Well-organised | To what extent is your community well organised in responding to the Coronavirus? | 6-point scale from 1(not at all) to 6 (very much) |
| Perceived fairness | Agree or disagree: - Not a lot is done for people like me in this country | 5-point scale from -2 (strongly disagree) to 2 (strongly agree) |

**Supplementary Figure S1**

*Directed acyclic graph of three research aims and corresponding statistical approaches*


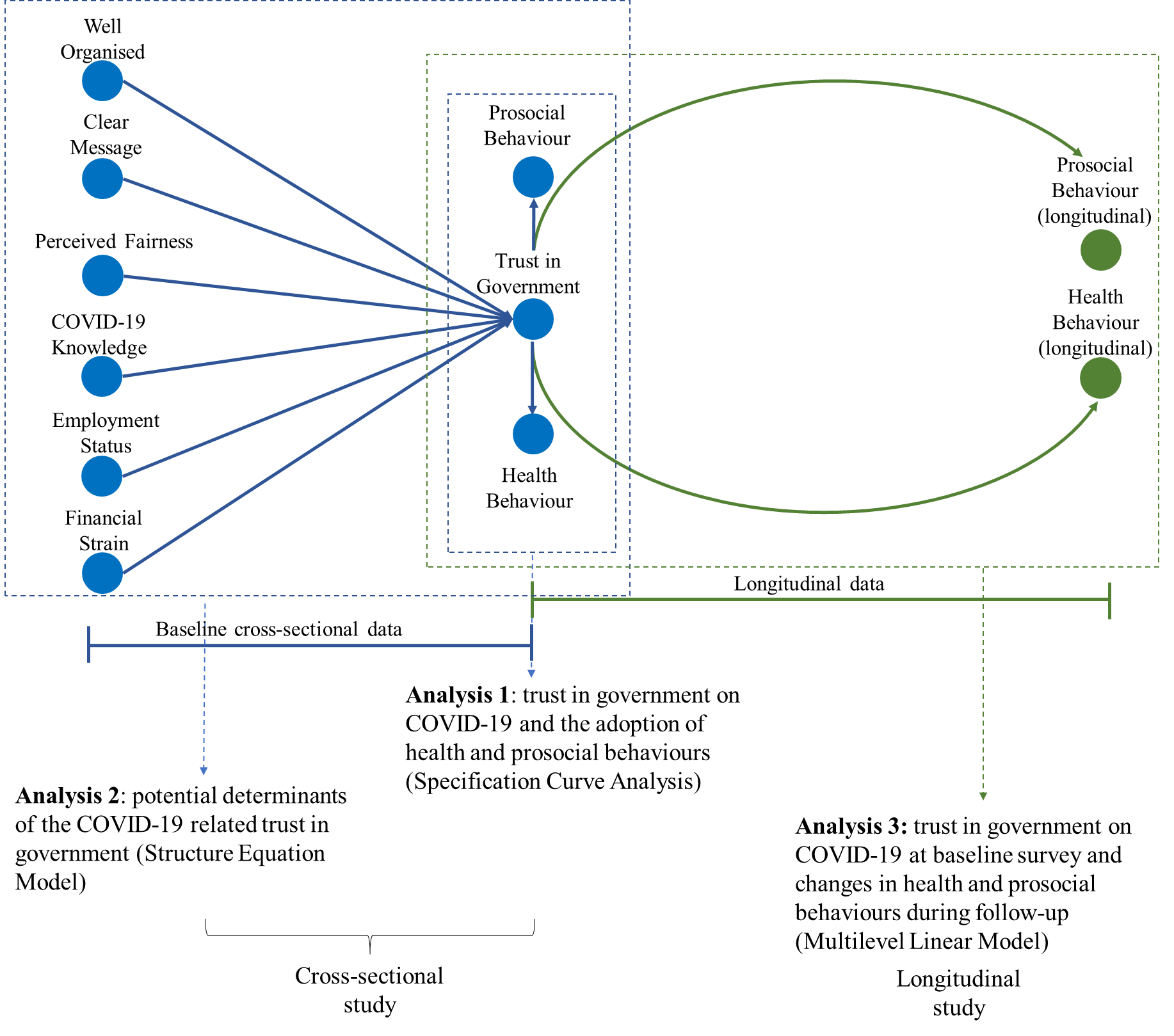

Supplement: Supplementary file 1 [file S0033291721001306sup001.docx]
